# Analysis of progression after elective distal ureterectomy and effects of salvage radical nephroureterectomy in patients with distal ureteral urothelial carcinoma

**DOI:** 10.1038/s41598-024-54232-4

**Published:** 2024-02-12

**Authors:** Chung Un Lee, Jong Hoon Lee, Hye Won Lee, Jae Hoon Chung, Wan Song, Minyong Kang, Hwang Gyun Jeon, Byong Chang Jeong, Seong Il Seo, Seong Soo Jeon, Hyun Hwan Sung

**Affiliations:** 1 Department of Urology, Chung-Ang University Gwangmyeong Hospital, Chung-Ang University College of Medicine, Gwangmyeong, Gyeonggi-do Republic of Korea; 2 Department of Urology, Samsung Medical Center, Sungkyunkwan University School of Medicine, 81 Irwon-ro, Gangnam-gu, Seoul, 06351 Korea

**Keywords:** Distal ureter, Distal ureterectomy, Progression, Radical nephroureterectomy, Therapeutic outcome, Urothelial carcinoma, Urology, Urological cancer

## Abstract

We compared the progression patterns after radical nephroureterectomy (RNU) and elective distal ureterectomy (DU) in patients with urothelial carcinoma of the distal ureter. Between Jan 2011 and Dec 2020, 127 patients who underwent RNU and 46 who underwent elective DU for distal ureteral cancer were enrolled in this study. The patterns of progression and upper tract recurrence were compared between the two groups. Progression was defined as a local recurrence and/or distant metastasis after surgery. Upper tract recurrence and subsequent treatment in patients with DU were analyzed. Progression occurred in 35 (27.6%) and 10 (21.7%) patients in the RNU and DU groups, respectively. The progression pattern was not significantly different (*p* = 0.441), and the most common progression site was the lymph nodes in both groups. Multivariate logistic regression analysis revealed that pT2 stage, concomitant lymphovascular invasion, and nodal stage were significant predictors of disease progression. Upper tract recurrence was observed in nine (19.6%) patients with DU, and six (66.7%) patients had a prior history of bladder tumor. All patients with upper tract recurrence after DU were managed with salvage RNU. Elective DU with or without salvage treatment was not a risk factor for disease progression (*p* = 0.736), overall survival (*p* = 0.457), cancer-specific survival (*p* = 0.169), or intravesical recurrence-free survival (*p* = 0.921). In terms of progression patterns and oncological outcomes, there was no difference between patients who underwent RNU and elective DU with/without salvage treatment. Elective DU should be considered as a therapeutic option for distal ureter tumor.

## Introduction

Upper tract urothelial carcinoma (UTUC) is a relatively uncommon urological cancer with a worldwide reaches 5–10% of urothelial cancers^1,2^. As a treatment for non-metastatic UTUC in ureter, radical nephroureterectomy (RNU) with bladder cuffing is known as the gold standard, but a single kidney status that occurs after surgery might causes renal insufficiency, dialysis, cardiovascular morbidity, and overall mortality^3–6^. For this reason, nephron-sparing surgery, such as segmental ureterectomy, has been considered mainly in imperative cases, such as patients with a single kidney or chronic kidney disease. Thus, there have been few studies that have only focused on elective segmental ureterectomy cases, not imperative cases.

Most UTUC tumors are located in the renal pelvis. Ureter tumors are much rarer, but their frequency has increased over the past 50 years and is expected to account for 25%–33% of UTUCs^7–9^. Distal ureterectomy (DU) or endoscopic ablation is recommended as a treatment method for distal ureteral tumors, but the level of evidence is low, and most of this evidence is based on imperative cases due to their rarity^2,10^. The oncological outcome of DU is not inferior to that of RNU and is advantageous for renal function^11–16^. Our team has previously reported similar results for DU in terms of oncological outcomes and renal function^11^. However, this outcome might be of limited significance, as 30% of the patients were imperative cases.

The probability of disease progression after RNU is reported to be 20–30%^17–20^, and the distribution of metastases after RNU is reported to be mostly in the lungs, liver, bone, and lymph node^21^. In particular, it is important to know whether elective DU and RNU have different patterns of progression and oncological outcomes when counseling patients before surgery and when explaining postoperative examinations and the risk of recurrence. To our knowledge, the patterns of disease progression and upper tract recurrence following elective DU, as well as the outcomes of salvage nephroureterectomy in distal ureter UC have been poorly reported. Therefore, this study aimed to compare the pattern of disease progression between RNU and elective DU and to analyze upper tract recurrence and subsequent salvage treatment in elective DU cases.

## Results

### Baseline characteristics

Between 2011 and 2020, 173 patients with distal ureteral urothelial carcinoma (UC) were enrolled. Among them, 127 patients underwent RNU and 46 patients underwent elective DU for UTUC in the distal ureter. As shown in Table 1, more patients who underwent RNU had diabetes mellitus as the underlying disease (*p* = 0.042). There were differences in the year of surgery (*p* < 0.001) and surgical approach (*p* < 0.001), but there were no differences in other baseline characteristics between the two groups (*p* > 0.05). The tumor size in the DU group was smaller than that in RNU group (3.1 ± 1.8 vs. 1.7 ± 1.0 cm, *p* < 0.001). Pathological characteristics, including T stage, tumor grade, concomitant lymphovascular invasion (LVI), lymph node (LN) stage, and margin status, were not significantly different between the groups (*p* > 0.05).

**Table 1:** Baseline characteristics between the RNU and DU groups.

	RNU group (*n* = 127)	DU group (*n* = 46)	*p* value
No. of patients	127	46	
Age, year	66.8 ± 9.6	64.8 ± 9.9	0.226
Sex, male, %	98 (77.2)	39 (84.8)	0.276
Body mass index	24.0 ± 2.9	24.5 ± 3.2	0.372
DM, %	39 (30.7)	7 (15.2)	**0.042**
HTN, %	59 (46.5)	22 (47.8)	0.873
Year of surgery, %			** < 0.001**
2011–2014	49 (38.6)	11 (23.9)	
2015–2017	29 (22.8)	25(54.3)	
2018–2020	49 (38.6)	10 (21.7)	
History of previous bladder cancer, %	34 (26.8)	14 (30.4)	0.637
Preoperative ureteroscopic exam, %	91 (71.7)	31 (67.4)	0.587
Approach type of surgery, %			** < 0.001**
Open	32 (25.2)	27 (58.7)	
Laparoscopic	81 (63.8)	0 (0)	
Robot-assisted	14 (11.0)	19 (41.3)	
Lymph node dissection, %	36 (28.3)	7 (15.2)	0.078
Risk stratification*, %			1
Low-risk	2 (1.6)	0 (0)	
High-risk	125 (98.4)	46 (100)	
Pathologic data			
Pathological T stage, %			0.480
Tis	2 (1.6)	3 (6.5)	
Ta	15 (11.8)	11 (23.9)	
T1	33 (26.0)	9 (19.6)	
T2	32 (25.2)	12 (26.1)	
T3	45 (35.4)	11 (23.9)	
Tumor grade			0.131
I, II	56 (44.1)	25 (54.3)	
III	69 (54.3)	18 (39.1)	
Concomitant LVI	20 (15.7)	5 (10.9)	0.420
Pathological N stage, %			0.186
Nx/N0	116 (91.3)	45 (97.8)	
≥ N1	11 (8.7)	1 (2.2)	
Margin positive, %	5 (3.9)	0 (0)	0.326
Tumor size, cm	3.1 ± 1.8	1.7 ± 1.0	** < 0.001**
Adjuvant Chemotherapy, %	38 (29.9)	7 (15.2)	0.051
Follow-up duration, months	53.3 ± 34.8	39.8 ± 21.4	**0.003**

Significant values are in [bold].

*Stratification based on European Association of Urology Guidelines 2023.

### Progression patterns between RNU and DU

There was no significant difference in progression patterns between the two groups (*p* = 0.441). Progression occurred in 35 patients (27.6%) in the RNU group and in 10 patients (21.7%) in the DU group. The most common site of progression was the lymph nodes in both groups (77.1% of the RNU group vs. 50% of the DU group). The pelvic cavity (31.4%), lung (20%), bone (11.4%), and liver (5.78%) followed in the RNU group. The lungs (20%), pelvic cavity (10%), and liver (10%) followed in the DU group (Table 2).

**Table 2:** Progression pattern between the RNU and DU groups.

	RNU group (*n* = 127)	DU group (*n* = 46)	*p* value
Progression	35 (27.6)	10 (21.7)	0.441
Lymph node	27 (77.1)	5 (50)	
Pelvic cavity	11 (31.4)	1 (10)	
Lung	7 (20.0)	2 (20)	
Bone	4 (11.4)	0 (0)	
Liver	2 (5.7)	1 (10)	
Kidney	0 (0)	1 (10)	
Adrenal gland	1 (2.9)	0 (0)	
Pancreas	1 (2.9)	0 (0)	
Colon	1 (2.9)	0 (0)	
Penis	0 (0)	1 (10)	
Vagina	1 (2.9)	0 (0)	
Multiple metastasis	13 (37.1)	1 (10)	

### Oncological outcomes including PFS, OS, CSS, and IVRFS between RNU and DU

**Figure 1:**
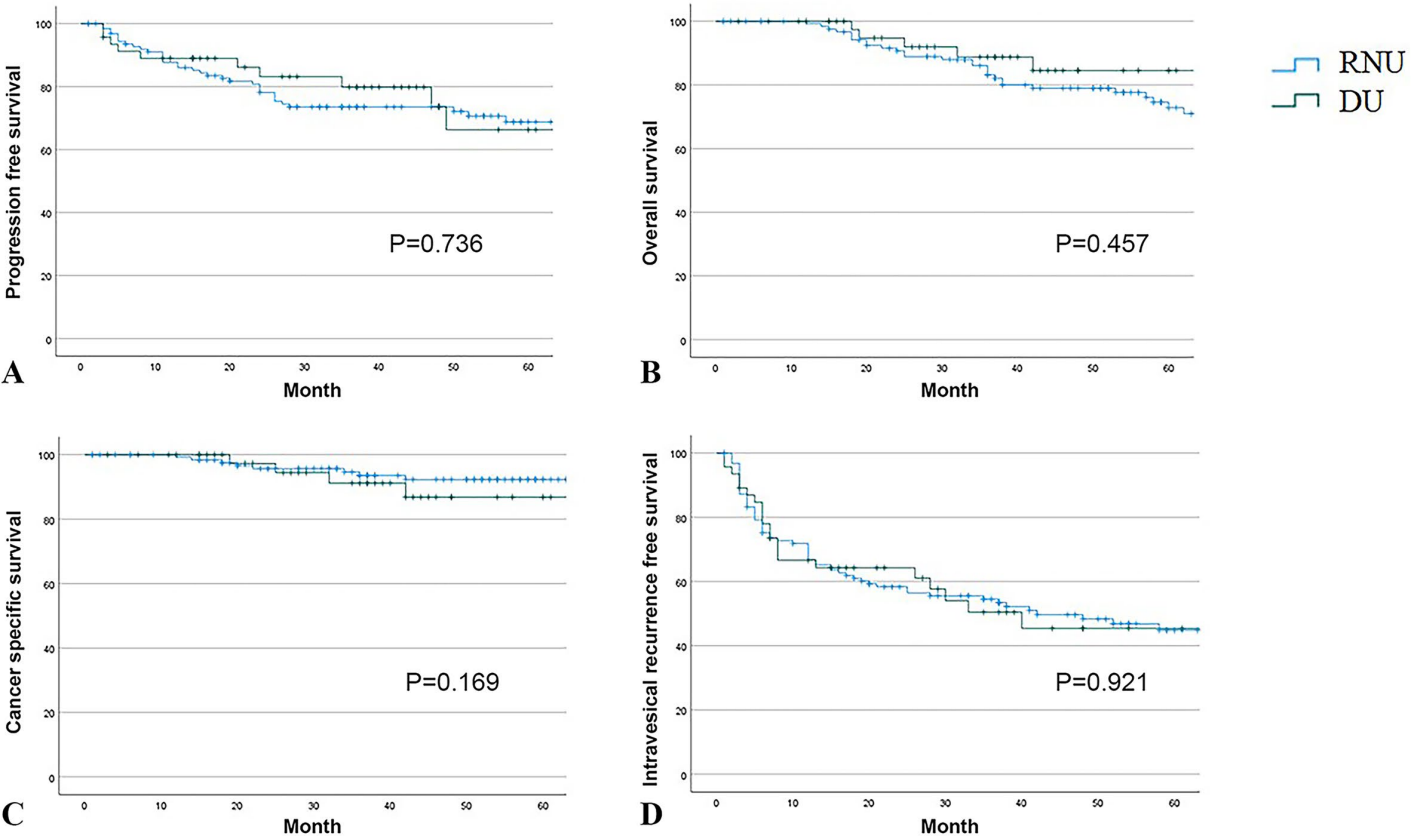
Kaplan–Meier analysis depicting (**A**) Progression free survival. (**B**) Overall survival. (**C**) Cancer specific survival; (**D**) Intravesical recurrence free survival between RNU and DU.

The 3-year PFS rates in the RNU and DU groups were 73.5% and 79.8%, respectively (*p* = 0.736; Fig. 1A). There were also no statistically significant differences in the 3-year OS and CSS rates between patients treated with RNU and DU (83.1% vs. 88.8%, *p* = 0.457, Fig. 1B; 93.6% vs. 91.2%, *p* = 0.169, Fig. 1C; respectively). There were no statistically significant differences in the 3-year IVRFS between patients treated with RNU and DU (54.5% vs. 50.4%, *p* = 0.921, Fig. 1D).

### Progression factors following surgery

Univariate analysis showed that the risk factors for progression after surgery included ≥ pT2, tumor grade III, concomitant LVI, and lymph node involvement (Hazard ratio (HR) 9.067, *p* < 0.001; HR 3.339, *p* = 0.002; HR 11.524, *p* < 0.001; and HR 41.088, *p* < 0.001, respectively). Independent predictors of progression in multivariate analysis were ≥ pT2, concomitant LVI, and LN involvement (HR 5.350, *p* = 0.005; HR 4.793, *p* = 0.006; and HR 23.454, *p* = 0.006, respectively). The surgical approach was not associated with progression (Table 3).

**Table 3:** Variables associated with risk of progression following surgery for distal ureter UC.

	Univariate			Multivariate		
	HR	95% CI	*p* value	HR	95% CI	*p* value
Age at surgery	0.971	0.938–1.006	0.102			
HTN	0.778	0.392–1.544	0.473			
DM	1.220	0.571–2.606	0.608			
BMI	0.994	0.885–1.116	0.916			
Prev. Bladder Ca	0.571	0.251–1.298	0.181			
T stage						
< pT2	Ref			Ref		
≥ pT2	9.067	3.361–24.459	** < 0.001**	5.350	1.644–17.416	**0.005**
Tumor grade						
I, II	Ref			Ref		
III	3.339	1.578–7.065	**0.002**	0.907	0.336–2.445	0.847
Concomitant LVI	11.524	4.379–30.326	** < 0.001**	4.793	1.585–14.493	**0.006**
N+	41.088	5.124–329.484	** < 0.001**	23.454	2.504–219.701	**0.006**
Margin	1.922	0.311–11.895	0.482			
Tumor size	1.091	0.906–1.312	0.358			
Surgical approach						
RNU	Ref			Ref		
DU	0.730	0.328–1.627	0.442	1.060	0.410–2.741	0.905

Significant values are in [bold].

### Upper tract recurrence pattern in patients with DU

Among 46 patients treated with DU, nine (19.6%) had recurred ipsilateral ureter or renal pelvis tumor during F/U duration of mean 18.3 ± 12.7 months. Of the nine patients, six (66.7%) had a prior history of bladder tumor. All patients underwent salvage RNU. Individual information regarding upper tract recurrence and management in the DU group is shown in Supplementary Table 1. In the univariate analysis, histories of bladder cancer history (HR 5.4, *p* = 0.035) and CIS (HR 18.000, *p* = 0.019) were significant predictors of upper tract recurrence following DU for distal ureteral cancer. However, in the multivariate analysis, there were no significant predictors of upper tract recurrence.

### Oncological outcomes including PFS, OS, CSS, and IVRFS between RNU, DU and DU with upper tract recurrence followed by salvage RNU

**Figure 2:**
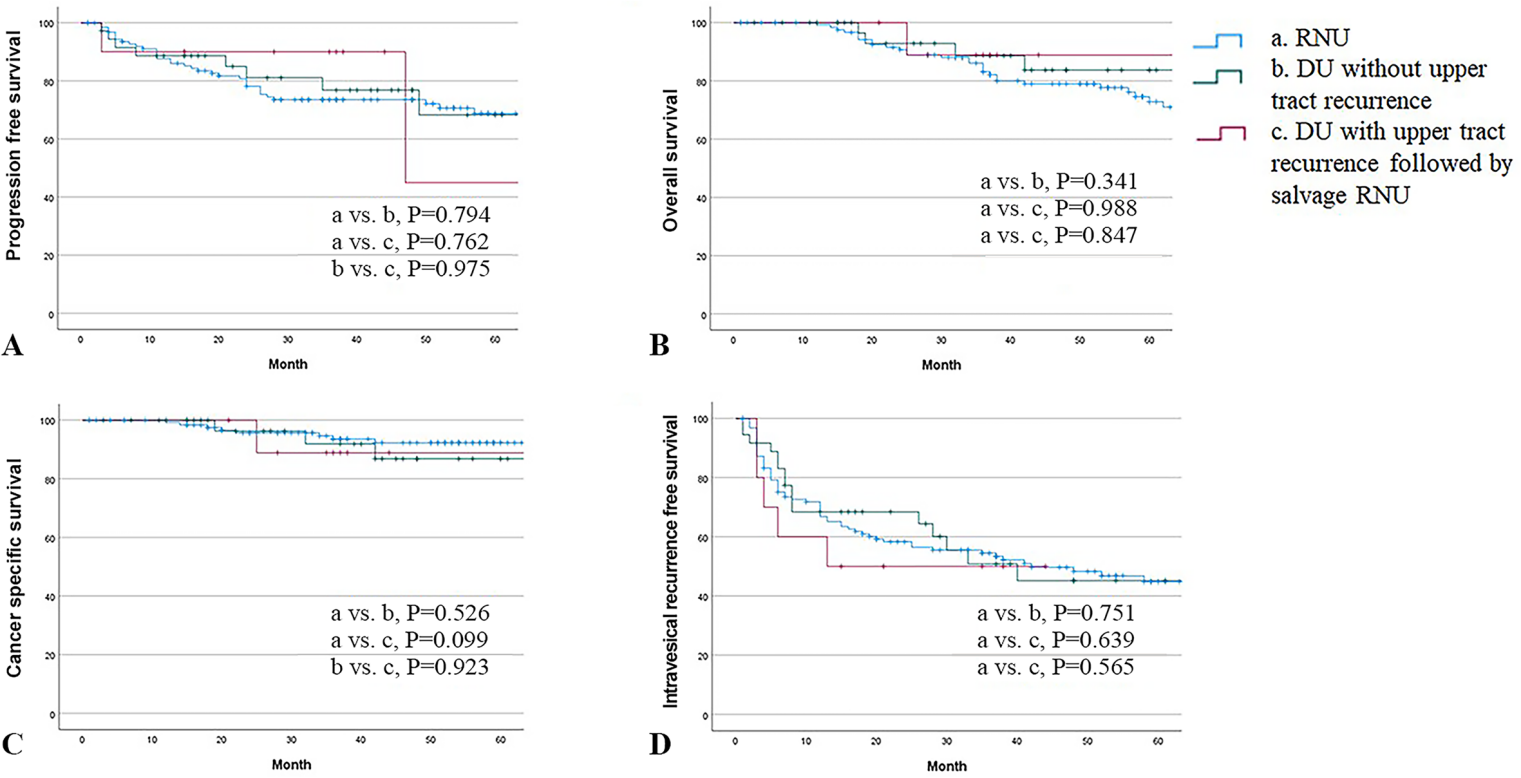
Kaplan–Meier analysis depicting (**A**) Progression free survival. (**B**) Overall survival. (**C**) Cancer specific survival. (**D**) Intravesical recurrence free survival between RNU, DU without upper tract recurrence and DU with upper tract recurrence followed by salvage RNU.

The 3-year PFS rates were 73.5%, 76.8%, and 90.0% in the RNU, DU without upper tract recurrence, and DU with upper tract recurrence followed by salvage RNU groups, respectively, which were not significantly different (*p* = 0.936, Fig. 2A). There were also no statistically significant differences in the 3-year OS and CSS rates among the three groups (83.1% vs. 88.6% vs. 88.9%, *p* = 0.673, Fig. 2B; 93.6% vs. 91.9% vs. 88.9%, *p* = 0.223, Fig. 2C; respectively). There were no statistically significant differences in 3-year IVRFS among patients treated with RNU, DU, or DU with upper tract recurrence, followed by salvage RNU (54.5% vs. 50.9% vs. 50.0%, *p* = 0.829, Fig. 2D).

### Functional outcomes between RNU and DU

Table 4 shows the changes in eGFR in the RNU and DU groups. As expected, patients treated with elective DU had significantly better eGFR preservation than those treated with RNU at 1, 3, and 6 months, and 1 year postoperatively (all *p* < 0.001).

**Table 4:** Comparison of preoperative eGFR and postoperative eGFR between the RNU and DU groups.

	RNU group	SU group	*p* value
Preoperative eGFR	64.0 ± 17.3	74.7 ± 19.2	< 0.001
Postoperative 1-month eGFR	53.0 ± 11.7	78.9 ± 15.4	< 0.001
Change in eGFR	− 11.0 ± 15.5	4.2 ± 12.0	< 0.001
eGFR preservation rate	86.9 ± 22.3	108.9 ± 22.6	< 0.001
Postoperative 3-month eGFR	51.7 ± 10.4	75.0 ± 13.4	< 0.001
Change in eGFR	− 12.5 ± 17.8	1.8 ± 15.2	< 0.001
eGFR preservation rate	85.7 ± 25.6	107.6 ± 35.5	< 0.001
Postoperative 6-month eGFR	49.0 ± 10.1	78.3 ± 14.7	< 0.001
Change in eGFR	− 14.3 ± 16.0	0.2 ± 21.1	< 0.001
eGFR preservation rate	81.8 ± 22.6	104.8 ± 29.6	< 0.001
Postoperative 1-year eGFR	50.5 ± 11.6	79.0 ± 16.3	< 0.001
Change in eGFR	− 12.6 ± 16.1	3.9 ± 15.0	< 0.001
eGFR preservation rate	84.0 ± 23.5	108.9 ± 27.6	< 0.001

## Discussion

This study aimed to compare the progression patterns after RNU and elective DU in patients with UC of distal ureter. A study on a similar topic was previously presented at our institute; there was no significant difference in oncologic outcomes between the RNU and segmental ureterectomy groups, and the functional outcome was superior in the segmental ureterectomy group. However, our previous study was limited because it only targeted imperative patients. Decreased renal function could lead worsening of the clinical course and outcome, and this makes it difficult to compare the progression after RNU and DU. Even if this fact is excluded, differences in progression between the two groups might exist. We conducted this study because we were concerned that the recurrence pattern of DU would be different from the gold standard treatment of RNU, when nephron sparing surgery was performed on patients with distal ureter tumor who do not have renal function problems, and there was no difference as a result analyzing through consecutive data with patients who underwent elective RNU and DU for distal ureteral urothelial carcinoma^11^. Additionally, we showed that patients who underwent RNU had decreased renal function, while patients who underwent DU had increased renal function.

Tanaka et al*.* reported the metastatic behavior of UTUC after RNU^21^. They reported that when the primary tumor was located in the lower ureter, the distant recurrence rate was 29.3%. The organ with the highest number of metastases was the liver, followed by the lungs, bones, and lymph nodes. In the present study, the progression rate after RNU for distal ureteral tumors was 27.6%, and the most common metastatic site was the lymph nodes, followed by the pelvic cavity, lungs, bone, and liver in the RNU group. Our study showed results similar to those of a previous study on metastatic patterns after RNU for distal ureteral cancer. On the other hand, Masson-Lecomte et al*.* reported the oncological outcomes of DU^22^. They reported that 71.9% of 5-yer OS, 84.4% of 5-year CSS, 74.4%, of 5-year homolateral upper tract recurrence free survival, and 43.6% of 5-year IVFS. Our study showed that 88.8% of 3-yer OS, 93.9% of 3-year CSS, 19.6% of homolateral upper tract recurrence, and 50.4% of 3-year IVFS, respectively.

Some studies have compared the oncological outcomes of DU with those of RNU. Giannarini et al*.* reported no significant difference in the 5-year OS and CSS between RNU and DU^23^, and Dalpiaz et al*.* reported equivalent oncologic control between the two groups^14^. In particular, Seisen et al*.* compared the oncologic outcomes between RNU and kidney-sparing surgery for distal ureters under elective conditions. They reported that 73.5% and 80.4%, 5-year CSS rates of 87.4% and 88.1%, and 5-year IVRFS rates of 46.7% and 53.4% of 5-year IVRFS rate in in the RNU and DU groups, respectively, with no significant difference^12^. In our study, 83.1% and 88.8%, and the 3-year CSS rates were 93.6% and 91.2% of 3-year CSS rate and 54.5% and 50.4% of 3-year IVRFS rate for the RNU and DU groups, respectively, with no significant difference between the two groups. Our results are consistent with those of previous studies.

Notably, the pathology of salvage RNU was quite similar to that of elective DU; thus, patients who underwent DU should be followed-up with rigorous surveillance, especially in cases with advanced-stage disease. Even upper tract recurrence after DU can be managed by salvage RNU. This is supported by our study, which found no significant difference in oncological outcomes between RNU, DU without upper tract recurrence, and DU with upper tract recurrence followed by salvage RNU. Upper tract recurrence is one of the most important and worrisome issues that must be taken into account during patient counseling prior to performing DU. Before surgery, it should be confirmed if it is a single lesion through imaging studies. If clinically possible, it is necessary to conduct a ureteroscopic exam before surgery to pass through the tumor and confirm whether there is another tumor above the target lesion. In this study, nine out of 46 (19.6%) cases had upper tract recurrence, which was not different from results of other studies. However, a significant number of upper tract recurrence cases (six, 66.7%) were related to prior bladder tumor history. Prior bladder cancer might be considered one of the clinically significant factors that cause upper tract recurrence following DU. In addition to the recurrence theories proposed in UC, the development of de novo upper tract tumors may be due to urinary reflux occurring after DU surgery. Since the anti-reflux mechanism will be lost when ureter reimplantation is performed after DU, patients with a previous history of bladder cancer are thought to be vulnerable to de novo upper tract tumor due to reflux. After DU surgery, immediate intravesical chemotherapy could be helpful to prevent de novo bladder tumor and upper tract recurrence although prosepctive randomized trials with risk stratification of bladder cancer are needed. If a distal ureter tumor has occurred in patients with a history of bladder cancer, sufficient counseling will be necessary when determining the treatment plan.

From a functional point of view, we assumed that preoperative renal function was already impaired due to obstruction of ureter in patients with distal ureter UC, and compensation was already underway in contralateral kidney. Therefore, in the RNU group, it is expected that there will be no significant difference in renal function even if the ipsilateral kidney is removed. Similarly, in DU group, obstruction was resolved, and renal function was thought to have improved.

Despite the strengths of this study, it has several limitations. First, the retrospective nature and non-randomized design might have contained significant selection bias. The smaller tumor size and shorter follow-up duration in the DU group were due to the retrospective design of the study. In addition, the surgical approach was based on the surgeon’s and/or patient’s preference, which might lead to another selection bias. Second, the population included in the study was not large, and the average follow-up period was less than 5 years, which was not sufficient to derive sufficient oncological results. Furthermore, due to the small number of patients, statistical analysis with adjustment for differences could not be performed. Third, we classified N0 and Nx into the same category, accounting for more than 90% of each group, which may have caused a bias in understanding.

In addition, there were differences in the surgical methods (open vs. laparoscopic vs. robotic) between two groups in our study. We previously reported that the robotic system has advantages for RNU^24^ and, especially when performing DU, is more beneficial in terms of the needlessness of an incision extension to remove the specimen. This is because robotic surgery has recently been performed, and DU has recently been performed using robotic systems. However, the differences between the surgical methods were not reflected.

## Conclusion

Progression patterns were similar in the RNU and elective DU groups, and the most common site of progression was the lymph nodes. Tumor stage, concomitant LVI, and nodal involvement were significant predictors of progression in patients with UTUC; however, the elective DU was not associated with cancer progression. Although upper tract recurrence frequently occurred in the DU group, it was safely managed with salvage RNU. To reduce upper tract recurrence and improve oncological outcomes, DU should be considered for patients without a history of bladder cancer. Physicians should counsel their patients before they undergo DU surgery. However, prospective trials should be conducted to confirm these findings.

## Materials and methods

### Study population and outcome parameters

This study was approved by the Institutional Review Board of the Samsung Medical Center (IRB No. 2022-11-031), which waived the requirement for informed consent owing to the retrospective nature of this study. All the study protocols were performed in accordance with the principles of the Declaration of Helsinki.

We retrospectively reviewed the records of patients who underwent RNU or elective DU for UTUC of the distal ureter between January 2011 and December 2020. In the case of elective DU, patients whose renal function was normal or who were likely to recover after the resolution of obstruction following surgery, even if their renal function was slightly decreased, were included. Patients who had a history of previous or concomitant radical cystectomy, had other malignancies, underwent RNU, had a history of previous DU, or underwent DU for imperative reasons, such as a single kidney, bilateral UTUC, or poor renal function, were excluded. Clinicopathologic characteristics, progression patterns, and oncological outcomes, including progression-free survival (PFS), overall survival (OS), cancer-specific survival (CSS), and intravesical recurrence-free survival (IVRFS), were compared between the two groups. Progression was defined as local recurrence and/or distant metastasis after surgery (upper tract and intravesical recurrence was excluded). Upper tract recurrence and salvage treatment after recurrence in patients with DU were analyzed. We also analyzed factors associated with progression after surgery for UTUC of the distal ureter.

To compare functional outcomes between RNU and DU, we additionally analyzed the preoperative estimated glomerular filtration rate (eGFR); specifically, postoperative 1-, 3-, 6- month eGFR and 1-year eGFR between RNU and DU. The eGFR was calculated from serum creatinine levels using the Modification of Diet in Renal Disease formula, which was adjusted for age and sex^25^.

### Surgical procedures

The selection of RNU or DU as treatment for tumors located in the distal ureter mainly depends on the surgeon and patient preference. Prior to the patient’s decision to undergo surgery, the surgeon provided sufficient information to the patients; RNU is the gold standard for current treatment, but DU has the advantage of preserving renal function and requires more examination, such as ureteroscopic examination, than RNU for evaluation because of the risk of recurrence in the ipsilateral ureter.

Regardless of the surgical approach, en bloc removal of the kidney, entire ureter, and bladder cuff was performed in RNU. A two-incision approach (flank incision for dissection of the kidney and ureter and Gibson incision for bladder cuff excision) was performed in open RNU. When performing laparoscopic RNU, the kidney and ureter were dissected maximally through the transperitoneal or retroperitoneal laparoscopic technique, and the bladder cuff was excised through a Gibson incision in the same manner as in the open technique. Robotic RNU were performed using a five-port transperitoneal approach with the four-arm da Vinci Si or Xi Surgical Systems (Intuitive Surgical, Sunnyvale, CA, USA). The port placement for robotic RNU with the da Vinci Si or Xi system was followed as previously described by the other center using a single-dock approach without intraoperative patient repositioning^26–28^. For DU, segmental resection of the distal ureter with bladder cuff excision and ureteral re-implantation were performed. In the case of open DU, the distal ureter and bladder cuff were resected en bloc through a low midline incision, and the remnant ureter was re-implanted through ureteroneocystostomy after repair of the open bladder. Robotic DU were performed using a six-port transperitoneal approach with the four-arm da Vinci Si or Xi Surgical Systems (Intuitive Surgical, Sunnyvale, CA, USA). The port placement for robotic DU with the da Vinci Si or Xi system was the same as that used for the robotic radical cystectomy previously described by the other center^29^. In principle, lymphadenectomy was performed in both RNU and DU; however, cases in which low stage and grade were estimated from preoperative images and ureteroscopic examinations were omitted.

### Follow-up regimen

In general, patients were followed up at 3- to 12-month intervals with cystoscopy, urine cytology, imaging of the upper tract collecting system and/or ureteroscopic examination, and specific work-up tools and intervals varied depending on the clinicians’ and patients’ preferences. Before performing DU, the clinicians fully explained to the patients that ureteroscopic examination under general anesthesia is necessary, and when patients agreed to that, ureteroscopic examination was performed at 3-to 12-month intervals with above examinations. Otherwise, CT scan was replaced. Upper tract recurrence was defined as tumor recurrence in the ipsilateral ureter of patients treated with DU.

### Statistical analyses

The primary outcome of this study was to compare the progression pattern between RNU and elective DU. The secondary outcomes were analyses of upper tract recurrence and salvage treatment after recurrence in patients with DU. Thus, the oncological outcomes of RNU, DU without upper tract recurrence, and DU with upper tract recurrence followed by salvage RNU were compared. Comparison of oncological outcomes, including PFS, OS, CSS, and IVRFS between RNU and DU, and progression factors following surgery were evaluated.

Descriptive statistics included the frequencies and proportions of categorical variables. Continuous variables are presented as means ± standard deviation for normally distributed data assessed using Student’s t-test. Categorical variables were compared using either the Pearson’s chi-square test or the stratified chi-square test. The Fisher’s exact test was used when appropriate. Kaplan–Meier survival analysis was used to illustrate the PFS, OS, CSS, and IVRFS of the two treatment groups. Logistic regression analysis was used to determine factors associated with progression. All statistical analyses were performed using IBM SPSS (version 27.0; SPSS Inc., Chicago, IL, USA), and statistical significance was defined as *p* < 0.05.

### Supplementary Information

Supplementary Information.

## Data Availability

Data that support the findings of this study are available upon reasonable request. If someone wants to request the data from this study, contact Chung Un Lee.

## Abbreviations

CSS: Cancer-specific survival
DU: Distal ureterectomy
eGFR: Estimated glomerular filtration rate
HR: Hazard ratio
IVRFS: Intravesical recurrence-free survival
LVI: Lymphovascular invasion
OS: Overall survival
PFS: Progression-free survival
RNU: Radical nephroureterectomy
UTUC: Upper tract urothelial carcinoma
