# Supplementary material for: Analysis of progression after elective distal ureterectomy and effects of salvage radical nephroureterectomy in patients with distal ureteral urothelial carcinoma
Source: Sci Rep. 2024 Feb 12;14:3497. doi: 10.1038/s41598-024-54232-4 (PMC10861547; doi:10.1038/s41598-024-54232-4)
Supplement: Supplementary file 1 — Supplementary Information. [file 41598_2024_54232_MOESM1_ESM.docx]

**Supplementary Table 1.** Upper tract recurrence and management for patients with DU

A. Summary of upper tract recurrence and management for patients with DU.

|  | **N** |
| --- | --- |
| **Upper tract recurrence, %** | 9 (19.6) |
| **Salvage RNU, %** | 9 (100) |
| **Mean duration from DU to upper tract recur** | 18.3±12.7 |
| **Prior bladder tumor history** | 6/9 (66.7%) |

B. Individual information of upper tract recurrence and management for patients with DU

| **No.** | **Age** | **Prior bladder tumor history** | **Duration (DU to UTUC recur), month** | **Salvage Treatment** | **Duration (DU to salvage treatment),**  **month** | **DU pathology** | **Salvage RNU pathology** | **Progression**  **(Duration – DU to progression, month)** | **Death**  **(Duration – DU to death, month)** | **Cause of death** |
| --- | --- | --- | --- | --- | --- | --- | --- | --- | --- | --- |
| **1** | 44 | Yes | 47 | Salvage RNU | 48 | T1G2Nx | T4G3N1 | Yes  (47) | Yes  (84) | UTUC |
| **2** | 65 | Yes | 4 | Salvage RNU | 6 | T2G3Nx, CIS | T2G2Nx | No | No |  |
| **3** | 58 | No | 12 | Salvage RNU | 12 | T3G3Nx | T3G3Nx | Yes  (15) | Yes  (25) | UTUC |
| **4** | 65 | No | 18 | Salvage RNU | 20 | T1G2Nx | T2G2Nx | No | No |  |
| **5** | 46 | Yes | 29 | Salvage RNU | 31 | T2G3Nx | T2G2N0 | No | No |  |
| **6** | 73 | Yes | 15 | Salvage RNU | 16 | T2G3Nx | T1G3Nx | No | No |  |
| **7** | 69 | Yes | 12 | Salvage RNU | 14 | CIS | T1G3N0, CIS | No | No |  |
| **8** | 62 | No | 11 | Salvage RNU | 13 | T3G3N0 | T3G3Nx | Yes  (15) | No |  |
| **9** | 77 | Yes | 17 | Salvage RNU | 22 | CIS | T1Nx, CIS | No | No |  |
